# Using Mössbauer Spectroscopy to Evaluate the Influence of Heat Treatment on the Surface Characteristics of Additive Manufactured 316L Stainless Steel

**DOI:** 10.3390/ma17143494

**Published:** 2024-07-15

**Authors:** Tatiana Ivanova, Michal Kořenek, Miroslav Mashlan

**Affiliations:** Department of Experimental Physics, Faculty of Science, Palacký University, 17. listopadu 12, 77900 Olomouc, Czech Republic

**Keywords:** selective laser melting, stainless steel, Mössbauer spectroscopy, X-ray powder diffraction, scanning electron microscopy, annealing, energy-dispersive analysis

## Abstract

The oxidation behaviour of iron-based 316L stainless steel was investigated in the temperature range of 700 to 1000 °C. The test specimens in the shape of plates were produced by selective laser melting. After fabrication, the samples were sandblasted and then annealed in air for different periods of time (0.5, 2, 8, 32 h). Under the influence of temperature and time, stainless steels tend to form an oxide layer. Scanning electron microscopy, energy dispersive analysis, and X-ray diffraction were employed to analyse the composition of this layer. Notably, a thin oxide layer primarily composed of (Fe-Cr) formed on the surface due to temperature effects. In addition, with increasing temperature (up to 1000 °C), the oxide of the main alloying elements, specifically Mn_2_(Fe-Cr)O_4_, appeared alongside the Fe-Cr oxide. Furthermore, the samples were subjected to conversion X-ray (CXMS) and conversion electron (CEMS) Mössbauer spectroscopy. CXMS revealed a singlet with a decreasing Mössbauer effect based on the surface metal oxide thickness. CEMS revealed the presence of Fe^3+^ in the surface layer (0.3 µm). Moreover, an interesting phenomenon occurred at higher temperature levels due to the inhomogeneously thick surface metal oxide layer and the tangential direction of the Mössbauer radiation towards the electron detector.

## 1. Introduction

Selective laser melting (SLM) is a subset of additive manufacturing (AM). The basic principle for producing complex three-dimensional models using 3D metal printing is the sequential melting of metal powder, layer by layer, using a heat source (laser, electron beam or arc) from computer-aided design (CAD) [1]. In SLM, a thin layer (20–100 µm) of metal powder is deposited on the moving platform and selectively melted by the laser beam. After the first layer is produced, the moving platform is lowered to a depth equal to the thickness of the first layer, a new layer of metal powder is added, and the process is repeated until the desired object is complete [2,3,4]. In recent years, the range of metal powders for AM has become much wider based on the requirements of the finished objects. Austenitic 316L stainless steels are widely used in many areas of industry [5,6] due to their excellent mechanical, physical and chemical properties [7,8,9].

Under the influence of high temperatures, stainless steel forms a protective layer of chromium oxide, due to the affinity of Cr for oxygen and the high content of this element in the chemical composition (approximately 20%). In general, surface oxidation is influenced by many factors, the most common of which are temperature and an oxidising atmosphere [10,11,12], the presence of alloying elements [13,14,15] and the type of surface treatment [16,17]. Recently, much research has been devoted to investigating the influence of temperature on surface behaviour. In [18,19], it was shown that temperature affects the resistance to diffusion of Cr, which increases the corrosion of austenitic steels. In [20], the authors investigated the effect of surface polishing on the formation of oxide layers with higher resistivity and Cr content. With regard to SLM, surface polishing is one of the most important aspects as it helps to remove the spherical particles from the surface of the finished product that remain after the laser process [21,22,23]. The austenitic phase is predominant in stainless steels; however, the transition from austenite to ferrite can occur under the influence of temperature. Temperature annealing is a necessity to relieve the internal stresses of the manufactured objects. The transition from austenitic to ferritic phase has been studied in [24,25], where austenitic stainless steel samples were annealed at different temperatures in an argon atmosphere [24] and in air [25]. In [26], the effect of alloying elements on the formation of martensite was investigated; the authors indicate that Cr is a ferrite-stabilising element, while Ni is an austenite-stabilising element. Both are predominant in the chemical composition of stainless steel.

Mössbauer spectroscopy [27] is an excellent tool for studying the surface of samples. The Mössbauer spectroscopy method has a high resolution, which makes it possible to record changes in the energy of nuclear transitions caused by the redistribution of electron density in the vicinity of the resonant nucleus and to obtain local features of the atomic environment in the lattice of a metal, alloy or compound. Most experiments are carried out on Fe alloys because this element is useful for observing the effect of resonance absorption of the gamma quantum isotope ^57^Fe. Mössbauer spectroscopy, together with X-ray diffraction, is a non-destructive method of studying materials and does not require any special sample preparation prior to measurement. Mössbauer spectroscopy uses the detection of conversion electrons and X-rays to study the surface and bulk of the samples.

The purpose of this research was therefore to carry out a study of oxides formed on the surface of 316L stainless steel under the influence of temperature, in order to trace the effect of temperature on the diffusion of atoms of alloying elements. For this purpose, the samples were produced by selective laser melting, sandblasted and annealed in air in the temperature range 700–1000 °C for 0.5, 2, 8 and 32 h. Conversion electron Mössbauer spectroscopy (CEMS) and conversion X-ray Mössbauer spectroscopy (CXMS) were used as instruments for bulk and surface studies. By studying the free path length of the electrons and the penetration depth of the X-rays, it is possible to obtain information about changes in the surface layers at depths of 0.3 µm and 10 µm, respectively. X-ray diffraction (XRD) was used to confirm the results obtained by Mössbauer spectroscopy, and the phase composition of the surface was studied as a reference. Scanning electron microscopy (SEM) provides information on the surface morphology of the samples. Energy-dispersive spectrometry (EDS) provides information on the localisation of the atoms of the elements in the alloy composition, depending on the depth of penetration of the electrons.

## 2. Materials and Methods

### 2.1. Materials and Temperature Treatment

In the present work, metal powder 316L (CL20ES) was purchased from Concept Laser. The approximate size of the spherical particles in the metal powder is 30 µm [28]. The chemical composition of the 316L powder is given in Table 1. For further experiment, metal plates with a size of 25 × 25 × 3 mm^3^ were prepared by the selective laser melting method using a Concept Laser M2-cusing system (GE Additive, Cincinnati, OH, USA). The SLM system consists of a Yb:YAG diode-pumped fibre optical laser with a wavelength of 1070 nm and a maximum power of 400 W. During laser operation, the laser power was set to 200 W and the maximum scanning rate was set to 1800 mm/s. The lower laser power was chosen on the basis of the author’s experience. After fabrication, the samples were sandblasted with corundum powder. The samples were subsequently annealed in air at 700–1000 °C. The temperature treatment was carried out for 0.5, 2, 8 and 32 h in a LE05/11 laboratory furnace (LAC, Židlochovice, Czech Republic). The sample was initially heated to the required temperature and then held in the furnace machine for a specified time. At the end of the holding time, the sample remained in the furnace until it had cooled completely.

### 2.2. Mössbauer Spectroscopy

The ^57^Fe Mössbauer spectra were measured at room temperature. For the accumulation of backscattering ^57^Fe Mössbauer spectra, a backscattering ^57^Fe Mössbauer spectrometer operating in constant acceleration mode and equipped with a ^57^Co(Rh) source and MS96 Mössbauer spectrometer software was used [29]. Spectra were recorded on 512 channels. For the registration of conversion X-ray Mössbauer spectra (CXMS), a proportional gas detector registering 6.4 keV X-rays was used, and an air scintillation detector was used for acquiring conversion electron Mössbauer spectra (CEMS) [30]. Least-squares fitting of the lines was used to calculate and evaluate the Mössbauer spectra using the MossWinn 4.0 software program [31,32]. The isomer shift values were related to the centroid of the spectrum recorded from an α-Fe foil (thickness 30 µm) at room temperature.

### 2.3. XRD Analysis, Surface Morphologya and Elemental Composition

XRD analysis provides information on the crystal structure and phase composition. Measurements were made on a Bruker Advance D8 X-ray diffractometer (Bruker, Billerica, MA, USA) with Bragg-Brentano parafocusing geometry. The diffractometer is equipped with a Co K_α_ X-ray source (wavelength 1.79026 Å, voltage 35 kV, current 40 mA) and a LYNXEYE position sensitive detector. On the primary beam path, the instrument was fitted with a 0.6 mm divergence slit and 2.5° axial Soller slits. On the secondary beam path, a 20 µm Fe K_β_ filter and 2.5° axial Soller slits were installed.

For surface imaging and elemental analysis using the EDS method, a scanning electron microscope VEGA3 LMU (TESCAN, Brno, Czech Republic) equipped with a secondary electron detector of Everhart-Thornley type (TESCAN, Brno, Czech Republic) and an XFlash silicon drift detector 410-M (Bruker Nano GmbH, Berlin, Germany) was used.

## 3. Results and Discussion

### 3.1. Surface Characterisationa and Oxide Morphology

**Figure 1:**
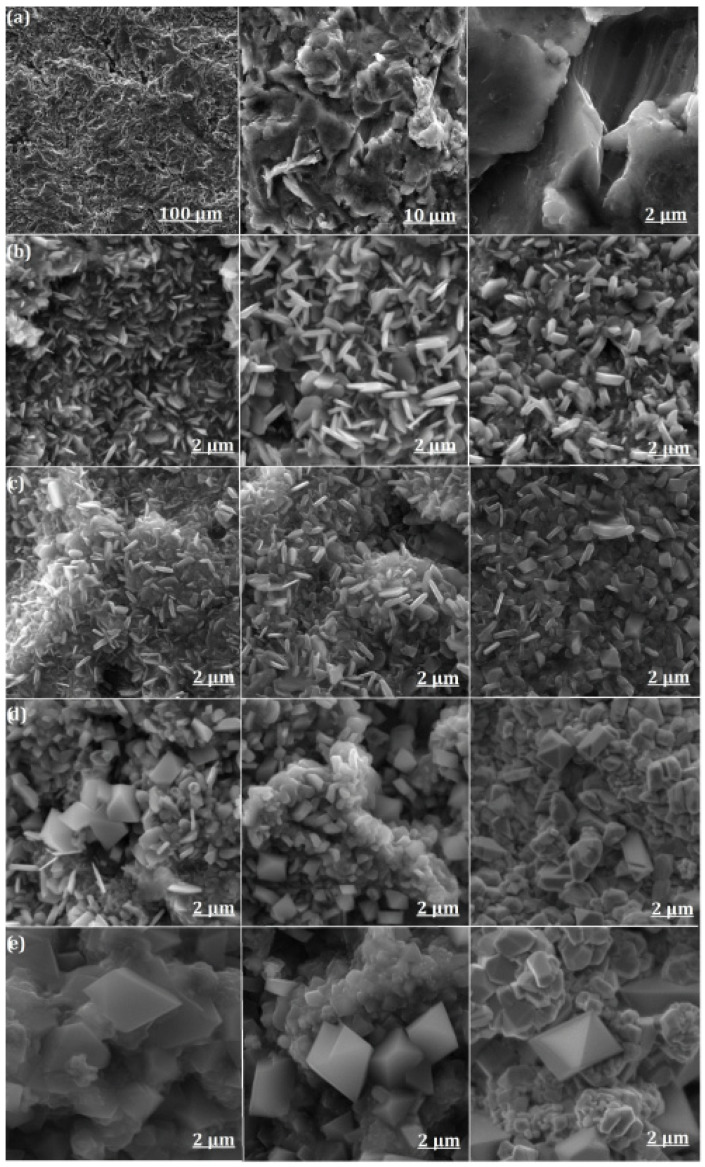
Surface morphology obtained from SEM after temperature impact (**a**) original sample surface, (**b**) 700 °C, (**c**) 800 °C, (**d**) 900 °C, (**e**) 1000 °C for 2 h (**left** column), 8 h (**middle** column) and 32 h (**right** column).

**Figure 2:**
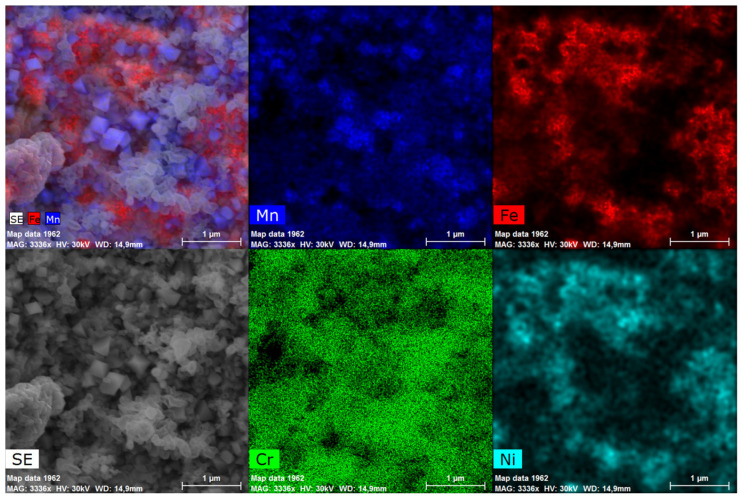
EDS mapping of the surface of the sample annealed at 1000 °C for 32 h.

Images of the morphology of the surface plane and oxides formed as a result of temperature exposure are shown in Figure 1. Under the initial influence of the temperature and air atmosphere, the surface is covered with an extensive layer of faceted metal oxide particles. Under the influence of a temperature of 700 °C on the sample surface, metal oxide growth begins with petal-shaped particles (Figure 1b). We have previously shown that petal-shaped particles correspond to an Fe-Cr mixed oxide [25], with point EDS indicating a dominant Cr content in these particles (Figure 3 in [25]). As previously reported, austenitic stainless steel tends to form the spinel-type oxide FeCr_2_O_4_ and a (Fe-Cr)_2_O_3_ mixed oxide when exposed to high temperatures and atmospheres [20,33,34,35,36]. All the annealing protocols used at 700 and 800 °C resulted in the formation of metal oxide flakes on the surface, presumably consisting of chromium oxide. In [35], the authors pointed out that the type of surface treatment and the exposure temperature influence the diffusion of Cr atoms from the bulk to the surface. The oxide layer can be described as a mixture of oxides of two elements, Fe and Cr. In [33], the authors found that stainless steel results in the formation of iron oxide on the surface of the sample because Fe ions have a greater tendency to diffuse than Cr ions. The oxide layer itself can be represented as a two-part structure, with the upper layer consisting of the Fe_2_O_3_ (hematite) and Fe_3_O_4_ (magnetite) and the spinel-type oxide FeCr_2_O_4_ or a (Fe-Cr)_2_O_3_ oxide below. With the effect of temperature, the 900 °C surfaces were enriched mainly in Cr with octahedral particles at the top, which increased with increasing time of the samples in the furnace (Figure 1d). At 1000 °C, octahedral particles appear on the surface of the samples studied. The size of the octahedral particles increases with increasing annealing time (Figure 1e). The visualisation of the EDS mapping shows that the octahedral particles contain Mn located on the surface (Figure 2).

**Figure 3:**
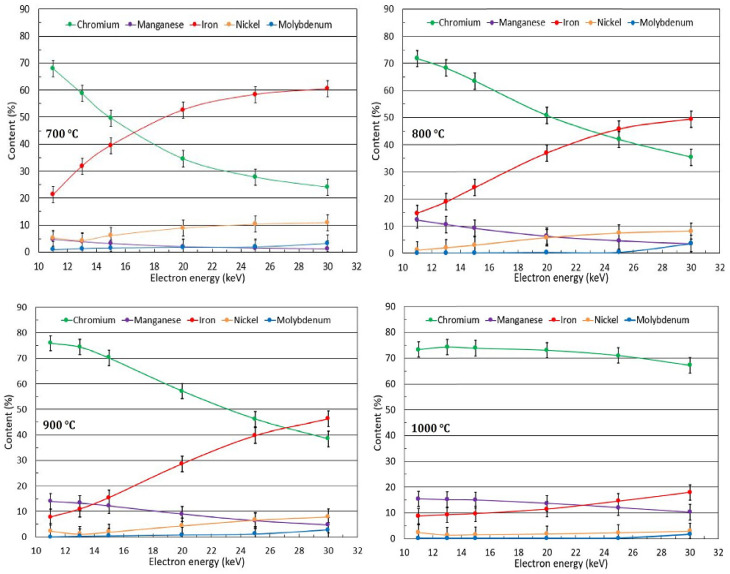
Depth dependence of element concentration of samples annealed at different temperatures for 32 h.

**Figure 4:**
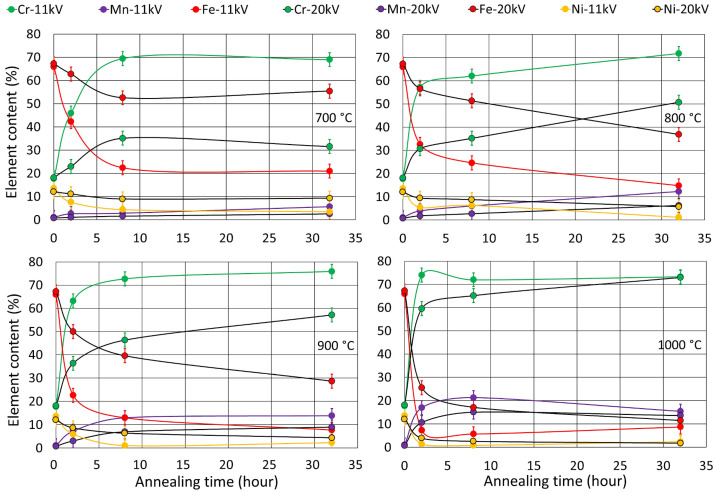
Dependence of elemental concentration for a sample annealed at 700–1000 °C for different times.

EDS analysis was used to determine the elemental composition at different depths. EDS data were obtained with primary electron acceleration voltages from 11 keV to 30 keV, corresponding to mass depths from 0.3 µm to 2.6 µm [37]. Figure 3 shows the depth elemental comparison results of the concentration in the samples at an annealing time of 32 h. According to the EDS analysis, the Cr content predominates at the surface, while the Fe content increases with depth or electron penetration. This is particularly evident in the sample annealed at 700 °C. By increasing the exposure temperature of the sample, the amount of Cr in the oxide layer increases, while the amount of Fe decreases significantly. In addition, at an annealing temperature of 800 °C, Mn growth is observed in the surface layer. During annealing at 900 °C and 1000 °C, the amount of Mn in the surface layer increases while the amount of Ni decreases. As a result of Mn diffusion from the bulk to the surface, the Mn_2_(Cr-Fe)O_4_-type oxide appears at the top of the sample. Figure 4 shows the dependence of the Fe and Cr in the sample on the hold time in the furnace at 700 ° C. According to the graph, Cr atoms are located on the surface of the oxide layer (0.3 µm), while Fe is distributed in the inner part. Probing at a depth of 2.5 µm shows that the Fe content is higher than the Cr content. Thus, the arrangement of the elements in the oxide layer shows that the Cr atoms are on the surface, while the Fe content is mostly in the bulk.

### 3.2. Oxide Phase Composition

**Figure 5:**
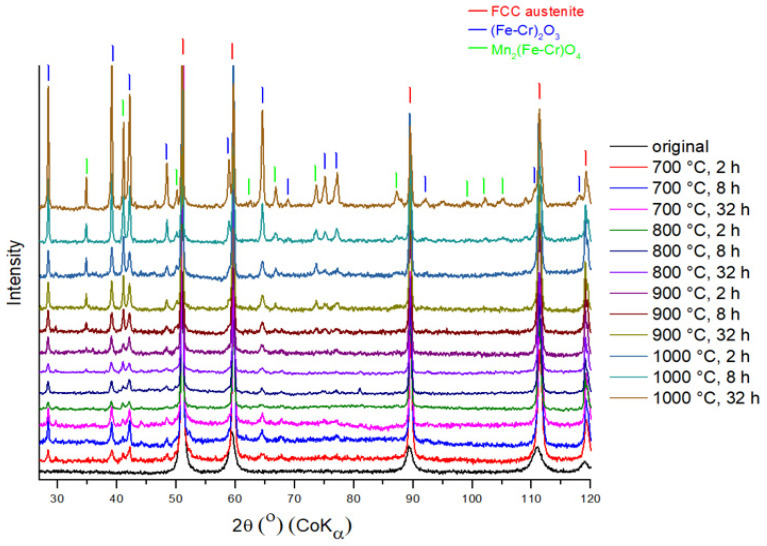
The XRD patterns of all stainless steel samples annealed in air in the temperature range 700–1000 °C.

The study of the phase changes in the samples as a result of the heat treatment was carried out using the method of XRD at room temperature. The XRD patterns of all the samples annealed in air atmosphere are shown in Figure 5. The Fe phases are further analysed by conversion MS in detail in Section 3.3.

The predominant phase in all samples is the austenitic phase. After temperature exposure, the oxide scale is observed on the XRD patterns. At an annealing temperature of 700 °C, the steel oxide scale on the surface consisted mainly of mixed oxide (Fe-Cr)_2_O_3_. In addition to the mixed phase, a Mn-containing oxide is formed on the surface. As the temperature increases, the peaks of the oxide phase become clearly visible. After a temperature exposure of 800 °C, the samples show a tendency to increase the oxide layer. A large volume fraction of the oxide layer is mixed oxide with a small amount of Mn oxide. At a holding time of 32 h, the proportion of Mn oxide increases, which may be due to the fact that the amount of Mn on the surface of the sample begins to increase. A significant increase in Mn oxide starts at an annealing temperature of 900 °C, which correlates with the data obtained from the EDS analysis. Annealing at 1000 °C results in a greater oxidation of the surface and, at a holding time of 32 h, it can be seen that the thickness of the oxide layer increases.

The formation of the mixed phase can occur due to the tendency of Cr atoms to diffuse under the influence of temperature [38]. Iron oxide (Fe_2_O_3_) and chromium oxide (Cr_2_O_3_) are difficult to distinguish on the XRD pattern due to phase overlap. Both oxides have the same crystal lattice type (trigonal) and the same crystallographic parameters. The diffusion coefficients of Mn and Fe are comparable and higher than those of Cr. Consequently, Mn and Fe have a high diffusion rate and easily diffuse into the oxide layer [39,40,41]. With increasing temperature, the appearance of a Mn-rich oxide has been observed in XRD patterns. In the composition of the initial metal powder, Ni is the second most common alloying element (Table 1). However, no Ni oxide was identified in the XRD patterns. This is in agreement with the elemental analysis by the EDS method (Figure 4), the Ni concentration in the surface layer did not increase after the initial samples were exposed to temperature. In [42], it was reported that Ni is less diffusive and is a slow diffusing element.

### 3.3. Mössbauer Spectroscopy

#### 3.3.1. Conversion X-ray Mössbauer Spectroscopy

**Figure 6:**
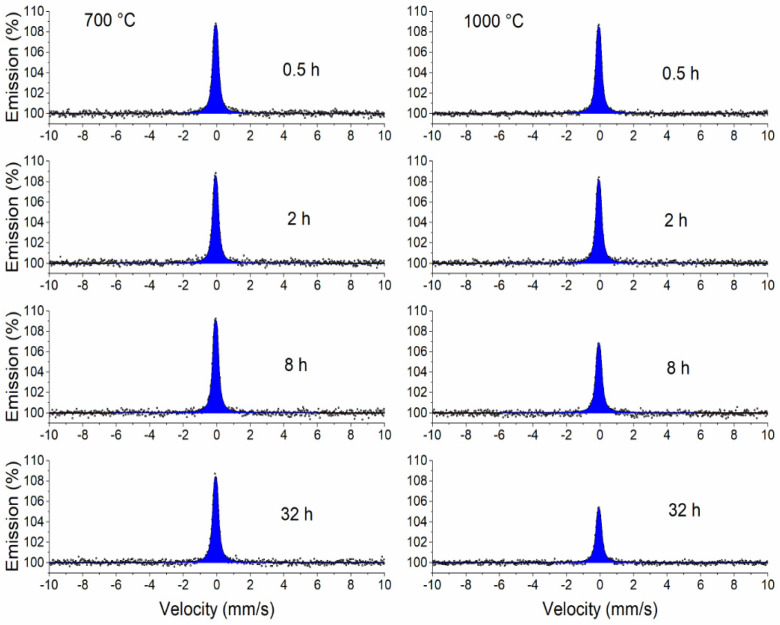
CXMS spectra of samples annealed at 700 and 1000 °C.

**Figure 7:**
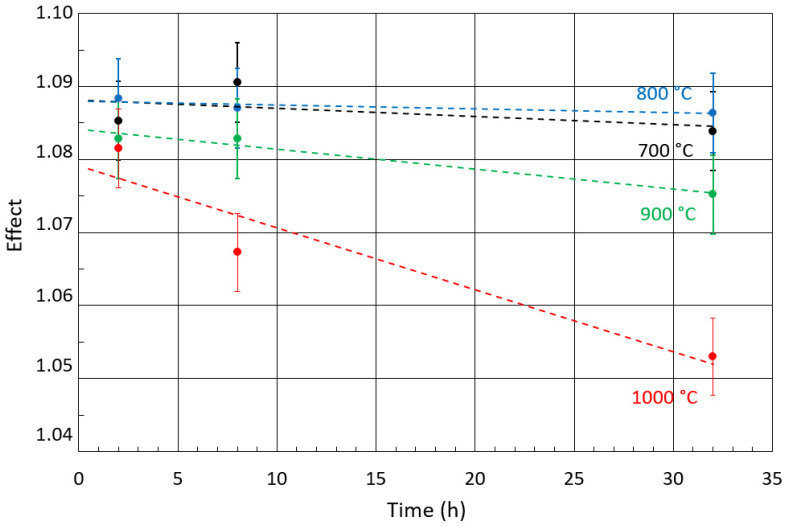
Magnitude of the Mössbauer effect (amplitude of the spectral line) in CXMS spectra for all samples.

CXMS was used to examine the surface layers of the annealed and unannealed samples to a depth of approximately 10 μm. Figure 6 shows only the Mössbauer spectra of the samples annealed at 700 and 1000 °C. The spectra of the samples annealed at 800 and 900 °C are not shown, because their shape is identical to the spectrum obtained at 700 and 1000 °C. All spectra are represented by a broad singlet corresponding to the FCC lattice. The spectra were measured in the same geometry, therefore the resulting intensity (spectral line amplitude) of the spectral lines reflects the attenuation of the resonant scattered radiation produced by the surface layer, the formation of which is mainly related to the diffusion and oxidation of Cr and Mn on the surface of the samples (see EDS and XRD results). Figure 7 shows that the thickness of the surface layer increases with temperature and annealing time. A model of a doublet with small quadrupole splitting was used to fit the spectra. The model used to fit the spectra represents Fe atoms arranged in a face-centred lattice. The small quadrupole splitting occurred due to the presence of alloying elements [43]. The results of the fitting using the MOSSWIN 4.0 software (Budapest, Hungary) for all CXMS spectra are presented in Table 2. The phase composition of the studied samples at a depth of 10 μm is the same as the phase composition of the original.

#### 3.3.2. Conversion Electron Mössbauer Spectroscopy

This method allows the study of a thin surface layer. The method is based on the emission of conversion electrons by resonance nuclei. The maximal diffusion depth of electrons in a material can be calculated using the formula [44]

(1) $$R=2.82\times 10^{-6}\frac{A{\left(E_{0}\right)}^{\frac{5}{3}}}{\rho {\left(Z\right)}^{\frac{8}{9}}}$$

where *E*_0_ is the maximal electron energy (keV), *ρ* is the density of matter (g/cm^3^), *A* is the atomic weight (g), *Z* is the atomic number and *R* is the diffusion depth (cm). According to this formula, the maximal probing depth into the Fe sample by CEMS is approximately 0.3 µm (maximal energy of the conversion electrons in ^57^Fe Mössbauer spectroscopy is 7.1 keV).

**Figure 8:**
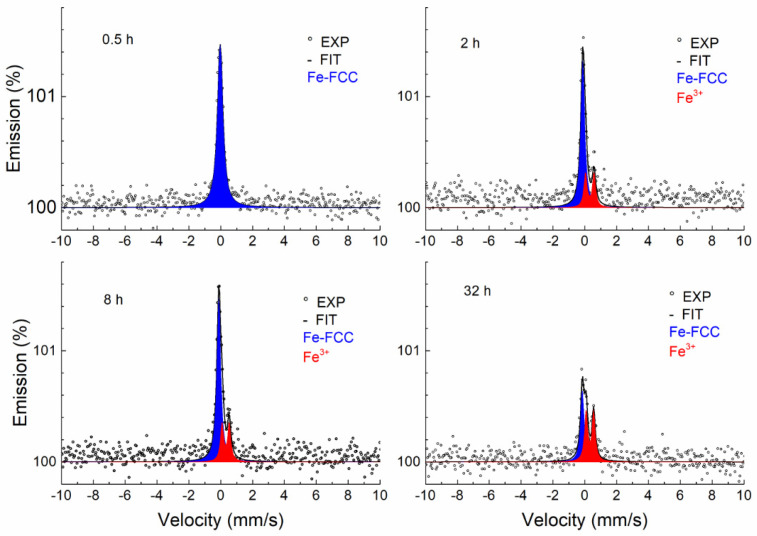
CEMS spectra of samples annealed at 700 °C.

**Figure 9:**
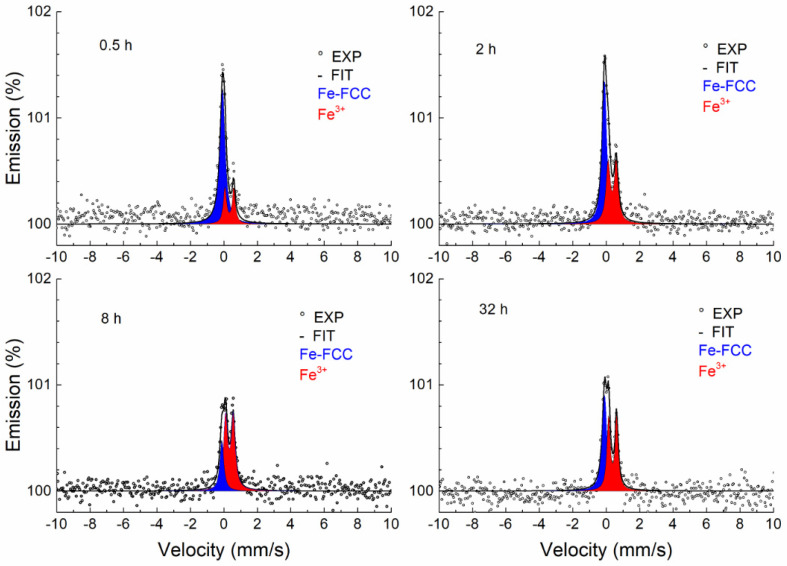
CEMS spectra of samples annealed at 800 °C.

**Figure 10:**
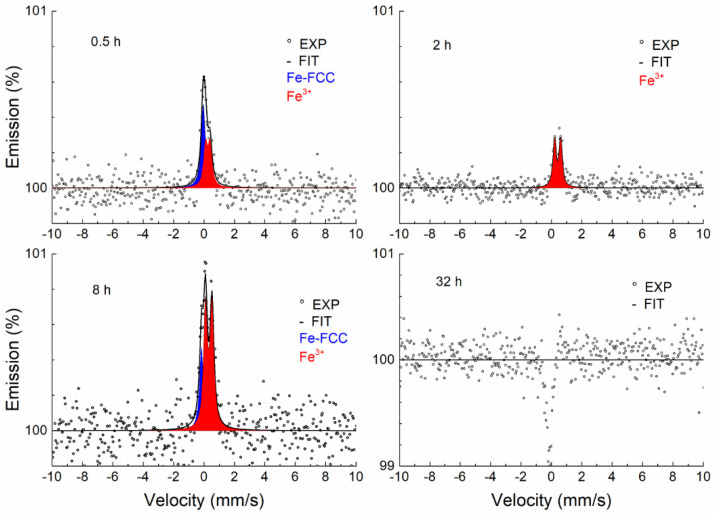
CEMS spectra of samples annealed at 900 °C.

**Figure 11:**
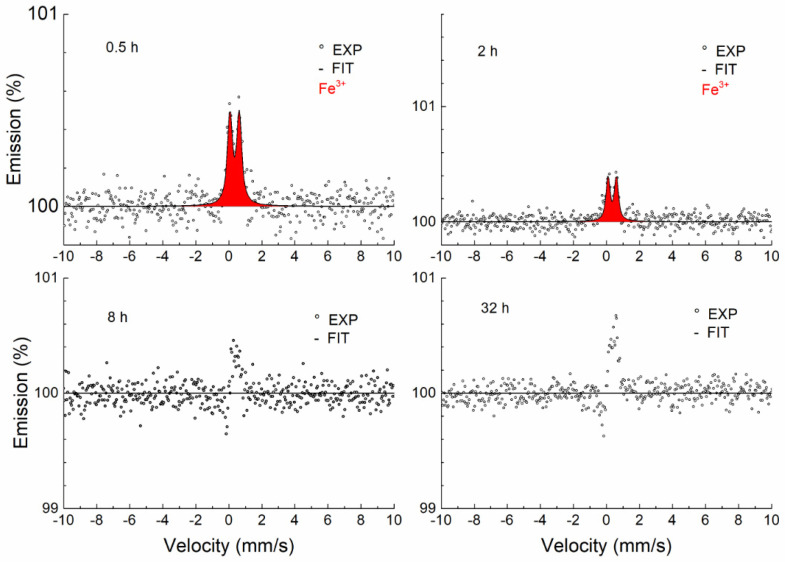
CEMS spectra of samples annealed at 1000 °C.

Figure 8 shows the CEMS spectra of samples annealed at a temperature of 700 °C for different periods of time. As can be seen, a broad singlet (blue) corresponding to the austenitic phase is present in all spectra, which is also characteristic of the annealed sample. Under the influence of the annealing time, a thin layer of Fe-containing oxide (Fe^3+^) begins to form on the surface of the sample. As the temperature increases, the thickness of the surface layer increases, which is reflected in a greater contribution of the Fe^3+^ doublet (red) (Figure 9, Figure 10 and Figure 11).

If the sample annealing time at a temperature of 900 °C is 2 h and at 1000 °C it is 0.5 or 2 h, the austenitic phase completely disappears and only a doublet corresponding to Fe-containing oxide can be seen. This effect may indicate that the sample is heterogeneous in the probing depth and alloying elements (Cr and Mn) accumulate on the thin surface layer, where together with Fe they form mixed oxides. The thickness of this layer is greater than 0.3 μm because we no longer see the austenitic phase in the spectrum.

A certain anomalous shape in the CEMS spectra was observed during annealing at 900 and 1000 °C and higher annealing times. The explanation for the unusual shape of the spectra is complicated and problematic. In our opinion, this is due to two facts. The first has to do with the type of electron detector [30] used, in which the Mössbauer radiation strikes tangentially on the surface of the sample. The second fact relates to the roughness of the studied surfaces. The record is probably distorted by a change in the background, which is partly composed of photoelectrons emitted via the photoeffect of Mössbauer radiation (14.4 keV). Because the sample surface is rough and the radiation is incident tangentially to the sample surface, part of the Mössbauer photons are resonantly absorbed by the ^57^Fe nuclei in the austenitic phase. The consequence is a reduction of the background in the resonance region. This phenomenon always occurs on a rough surface but does not always manifest itself. It was visible in cases where a heterogeneous layer with a low Fe content was formed on the surface of the sample. This has never been observed before; it is a combination of the properties of the electron detector used with the tangential direction of the Mössbauer radiation and the surface roughness of the sample being studied (Appendix A).

The results of the fitting using the MOSSWIN software for CEMS spectra are presented in Table 3. In addition to the austenitic phase, the Fe^3+^ phase was identified in a heterogeneous layer about 0.3 µm thick. The doublet corresponding to the austenitic phase in the annealed samples was fitted with the fixed parameters of the unannealed sample, the reason being the partial overlap with the doublet of the oxide phase (Fe^3+^). Due to the effect of surface roughness on the background spectra, the ratio of these phases cannot be quantitatively estimated. At the same time, the surface roughness effect is the cause of the dispersion of the oxide (Fe^3+^) phase doublet parameters.

The Fe^3+^ doublet was identified by CEMS in the surface layer (0.3 μm) in parts annealed in air at temperatures between 700 and 1000 °C. The Mössbauer parameters (Table 3) of the identified Fe^3+^ doublet cannot be identified with those of known iron oxides [45]. Cr and Mn oxides with partial iron substitution were identified by XRD. We can therefore conclude that the Fe^3+^ doublet observed by CEMS corresponds to one of the two substances (Fe-Cr)_2_O_3_ and Mn_2_(Cr-Fe)O_4_ identified by XRD. Since Mn_2_(Cr-Fe)O_4_ was observed in XRD patterns only at 1000 °C and (Fe-Cr)_2_O_3_ appeared also at lower temperatures, the Fe^3+^ doublet observed in CEMS can be assigned to (Fe-Cr)_2_O_3_.

## 4. Conclusions

The effect of temperature on the surface layer of stainless steel samples prepared by SLM was studied. Annealing was carried out in air in a temperature range of 700–1000 °C for varying lengths of time. A study of the surface of the steel parts has shown that annealing leads to the formation of an inhomogeneous surface layer that prevents deep oxidation. The surface morphology showed the presence of flake-shaped crystals of the trigonal mixed oxide (Fe-Cr)_2_O_3_. The number and size of the crystals increased with increasing annealing time. Starting with a temperature of 800 °C and a time of 32 h, additional particles of the octahedral-shape of Mn_2_(Fe-Cr)O_4_ appeared on the surface. With increasing temperature and time, the surface was completely covered with a layer of mixed oxides. Mössbauer spectroscopy allowed the material to be studied at different depths. According to the CXMS results, at a depth of 10 µm, only a wide singlet corresponding to the austenitic phase was detected. In the case of the CEMS data, at a depth of 0.3 µm, a singlet of the austenitic phase with the Fe containing doublet (Fe^3+^) occurred. In accordance with the XRD results, this doublet was assigned (Fe-Cr)_2_O_3_. With increasing exposure to temperature and annealing time, the thickness of the (Fe^3+^) oxide increased. As a result, after the impact of high temperatures (900 °C and 1000 °C), the austenitic phase completely disappeared. Some of the CEMS spectra after annealing at 900 °C and 1000 °C had an unusual shape. This unusual profile was explained by a combination of the use of an electron detector, the geometry of the measurement and the roughness of the sample surface. The recording was distorted due to a change in the background (in the austenite resonance), which is partly composed of photoelectrons emitted due to the photoeffect of Mössbauer radiation (14.4 keV).

## Figures and Tables

**Table 1:** Chemical composition of metal powder (material data Concept Laser GmbH).

Element Concentration, wt %								
Fe	C	Si	Mn	P	S	Cr	Mo	Ni
Balance	≤0.03	0–1.0	0–2.0	≤0.045	≤0.03	16.5–18.5	2.0–2.5	10.0–13.0

**Table 2:** Hyperfine parameters of CXMS spectra (IS—isomer shift, QS—quadrupole splitting, FWHM—full width at half maximum).

Temperature (°C)	Time (h)	Phase	IS (mm/s) ±0.01	QS (mm/s) ±0.01	FWHM (mm/s) ±0.01
Unannealed	-	Austenite	−0.10	−0.17	0.29
700 °C	0.5	Austenite	−0.10	−0.16	0.28
	2	Austenite	−0.10	−0.15	0.28
	8	Austenite	−0.10	−0.16	0.27
	32	Austenite	−0.10	−0.15	0.28
800 °C	0.5	Austenite	−0.08	−0.16	0.27
	2	Austenite	−0.10	−0.15	0.26
	8	Austenite	−0.10	−0.15	0.29
	32	Austenite	−0.10	−0.14	0.29
900 °C	0.5	Austenite	−0.08	−0.15	0.28
	2	Austenite	−0.08	−0.16	0.27
	8	Austenite	−0.08	−0.16	0.27
	32	Austenite	−0.10	−0.15	0.27
1000 °C	0.5	Austenite	−0.10	−0.15	0.27
	2	Austenite	−0.08	−0.15	0.28
	8	Austenite	−0.10	−0.16	0.29
	32	Austenite	−0.10	−0.14	0.29

**Table 3:** Hyperfine parameters of CEMS spectra (IS—isomer shift, QS—quadrupole splitting, FWHM—full width at half maximum).

Temperature, (°C)	Time, (h)	Phase	IS (mm/s) ±0.02	QS (mm/s) ±0.03	FWHM (mm/s) ±0.03
Unannealed	-	Austenite	−0.12	0.15	0.25
700 °C	0.5	Austenite	−0.12 *	0.15 *	0.25 *
	2	Austenite	−0.12 *	0.15 *	0.25 *
		Fe^3+^	0.33	0.57	0.25
	8	Austenite	−0.12 *	0.15 *	0.25 *
		Fe^3+^	0.33	0.48	0.24
	32	Austenite	−0.12 *	0.15 *	0.25 *
		Fe^3+^	0.36	0.48	0.25
800 °C	0.5	Austenite	−0.12 *	0.15 *	0.25 *
		Fe^3+^	0.32	0.52	0.26
	2	Austenite	−0.12 *	0.15 *	0.25 *
		Fe^3+^	0.35	0.49	0.31
	8	Austenite	−0.12 *	0.15 *	0.25 *
		Fe^3+^	0.34	0.43	0.26
	32	Austenite	−0.12 *	0.15 *	0.25 *
		Fe^3+^	0.38	0.49	0.24
900 °C	0.5	Austenite	−0.12 *	0.15 *	0.25 *
		Fe^3+^	0.21	0.30	0.29
	2	Fe^3+^	0.40	0.40	0.25
	8	Austenite	−0.12 *	0.15 *	0.25 *
		Fe^3+^	0.32	0.42	0.35
	32	-	-	-	-
1000 °C	0.5	Fe^3+^	0.33	0.58	0.36
	2	Fe^3+^	0.36	0.52	0.28
	8	-	-	-	-
	32	-	-	-	-

* fixed parameter.

**Figure A1:**
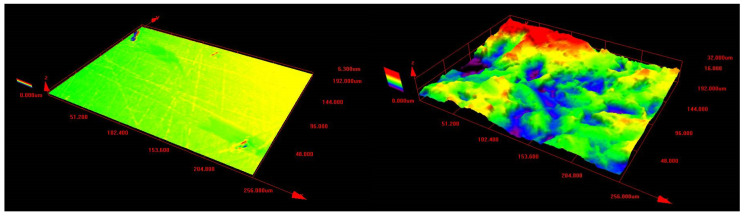
Surface roughness of polished (**left**) and unpolished (**right**) surfaces after annealing at 1000 °C for 32 h.

**Figure A2:**
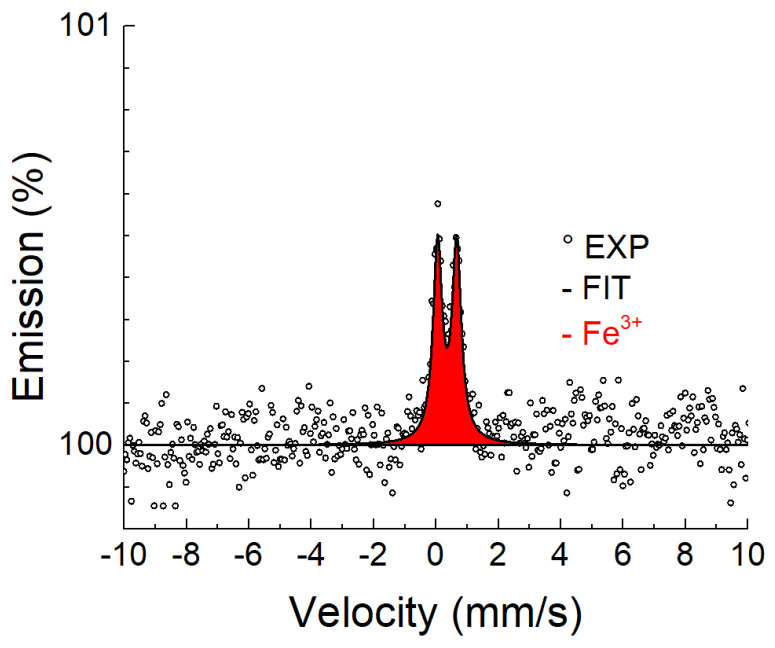
CEMS spectrum of a polished sample annealed at 1000 °C for 32 h.

## Data Availability

The original contributions presented in the study are included in the article, further inquiries can be directed to the corresponding authors.

## Appendix A

The hypothesis of the influence of the surface roughness of the studied sample on the CEMS spectra was additionally experimentally confirmed by studying a sample with a polished surface. The surface of the unannealed sample was polished on a metallographic polisher and then the sample was annealed at 1000 °C for 32 h. The surface roughness was evaluated using a confocal microscope (LEXT). Figure A1 shows the surface of the polished and unpolished specimen. Longitudinal roughness R_a_ and surface roughness S_a_ were calculated for both polished and unpolished samples (Table A1).

**Table A1:** Roughness of polished and unpolished surfaces after annealing at 1000 °C for 32 h.

Sample	R_a_	S_a_
polished	0.07 ± 0.02	0.08
unpolished	2.64 ± 0.37	3.44

The CEMS spectrum of a polished sample annealed at 1000 °C for 32 h is shown in Figure A2. It can be seen that it consists only of a doublet corresponding to the oxide phase (Fe^3+^). The spectrum does not contain an austenitic phase nor is there any obvious deformation of the spectrum, i.e., a background change related to the resonance absorption of Mössbauer radiation (14.4 keV) by the rough surface protrusions.
